# A Rare but Lethal Emergency: A Case Report on Boerhaave’s Syndrome

**DOI:** 10.7759/cureus.80377

**Published:** 2025-03-10

**Authors:** Girish Bakhshi, Shivangi Tiwari, Aishwarya Dutt, Balakrishna Menon, Bhagyashree Ochiramani

**Affiliations:** 1 General Surgery, Grant Government Medical College and Research Institute, Mumbai, IND; 2 General Surgery, Grant Government Medical College, Mumbai, IND

**Keywords:** boerhaave's syndrome, emergency thoracotomy, increased intraesophageal pressure, multidisciplinary approach, muscle flap repair

## Abstract

Boerhaave's syndrome is a rare and life-threatening condition characterized by spontaneous esophageal rupture, often precipitated by sudden increases in intra-esophageal pressure, typically following forceful vomiting. The nonspecific nature of its clinical presentation, which may include chest pain, vomiting, and dyspnea, often overlaps with other thoracic or gastrointestinal disorders, making diagnosis challenging. This case report describes a 71-year-old female who presented with acute chest pain following episodes of vomiting. Initial clinical examination revealed tachycardia, tachypnea, and subcutaneous emphysema, raising suspicion of Boerhaave's syndrome. Diagnosis was confirmed through contrast-enhanced computed tomography (CECT), which revealed a 3 cm perforation in the thoracic esophagus with pleural effusion.

The patient was managed surgically with emergency thoracotomy, debridement, primary esophageal repair reinforced with an intercostal muscle flap, and the placement of a feeding jejunostomy. This case underscores the importance of early diagnosis, prompt surgical intervention, and multidisciplinary management in mitigating the high mortality associated with Boerhaave's syndrome.

## Introduction

Boerhaave's syndrome, first documented in 1724, denotes a rare yet critical medical condition characterized by the spontaneous rupture of the esophagus. This syndrome typically arises from a sudden and intense surge in intra-esophageal pressure, often triggered by forceful vomiting or retching [1]. Consequently, the rupture allows gastric contents to escape into the mediastinal and pleural spaces, potentially leading to severe complications like mediastinitis, sepsis, and respiratory compromise.

Despite notable advancements in medical technology and diagnostic methodologies, the diagnosis of Boerhaave's syndrome remains challenging. A primary obstacle stems from the nonspecific nature of its clinical presentation [2]. Patients frequently present with symptoms such as acute chest pain, vomiting, dyspnea, and subcutaneous emphysema. However, these symptoms can overlap with those of various other gastrointestinal and thoracic disorders, thereby impeding prompt diagnosis and treatment initiation.

The present case involves a 71-year-old female who presented with complaints of chest pain following episodes of vomiting. After the diagnosis of Boerhaave's syndrome with the help of radiological investigations such as contrast-enhanced computed tomography (CECT), the patient was managed by adequate drainage and control of sepsis with close intensive care monitoring followed by thoracotomy and debridement, which is the cornerstone for the treatment of Boerhaave's syndrome.

## Case presentation

A 71-year-old female patient presented with sudden and severe chest pain accompanied by episodes of vomiting after eating. The patient took over-the-counter antacids to relieve the pain; however, the pain was unbearable, which prompted a visit to the hospital. Upon clinical examination, the patient exhibited tachycardia (pulse: 110/min) and tachypnea (respiratory rate: 26/min). Abdominal examination revealed a soft, non-tender abdomen, with no signs of guarding or rigidity. An electrocardiogram was suggestive of sinus tachycardia.

**Figure 1:**
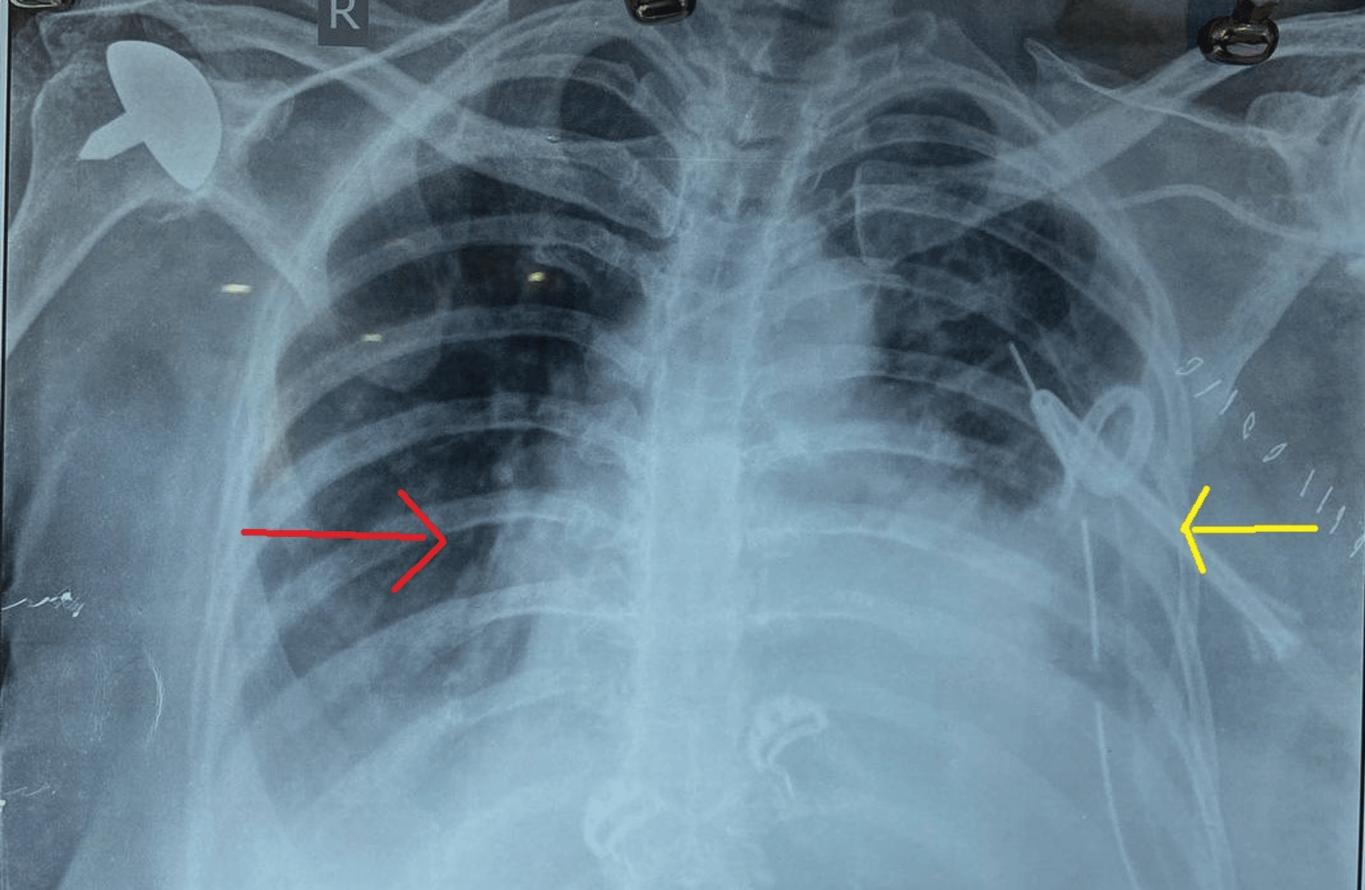
Chest radiograph depicting left-sided pleural effusion (yellow arrow) with subcutaneous emphysema (red arrow)

Upon admission to the hospital, intravenous analgesics and proton pump inhibitors were administered, which provided partial relief, followed by persistent, unbearable epigastric and chest pain radiating to the back. Chest X-ray showed left side pleural effusion (Figure 1). On clinical examination after six hours, the patient developed subcutaneous emphysema in the anterior chest wall.

The identification of pneumomediastinum, subcutaneous emphysema, and left-sided pleural effusion on the chest radiograph raised concerns for Boerhaave's syndrome. Given the persistence of her symptoms and ongoing suspicion for an underlying pathology, following initial resuscitation, a CECT scan of the chest and abdomen with oral contrast was performed.

**Figure 2:**
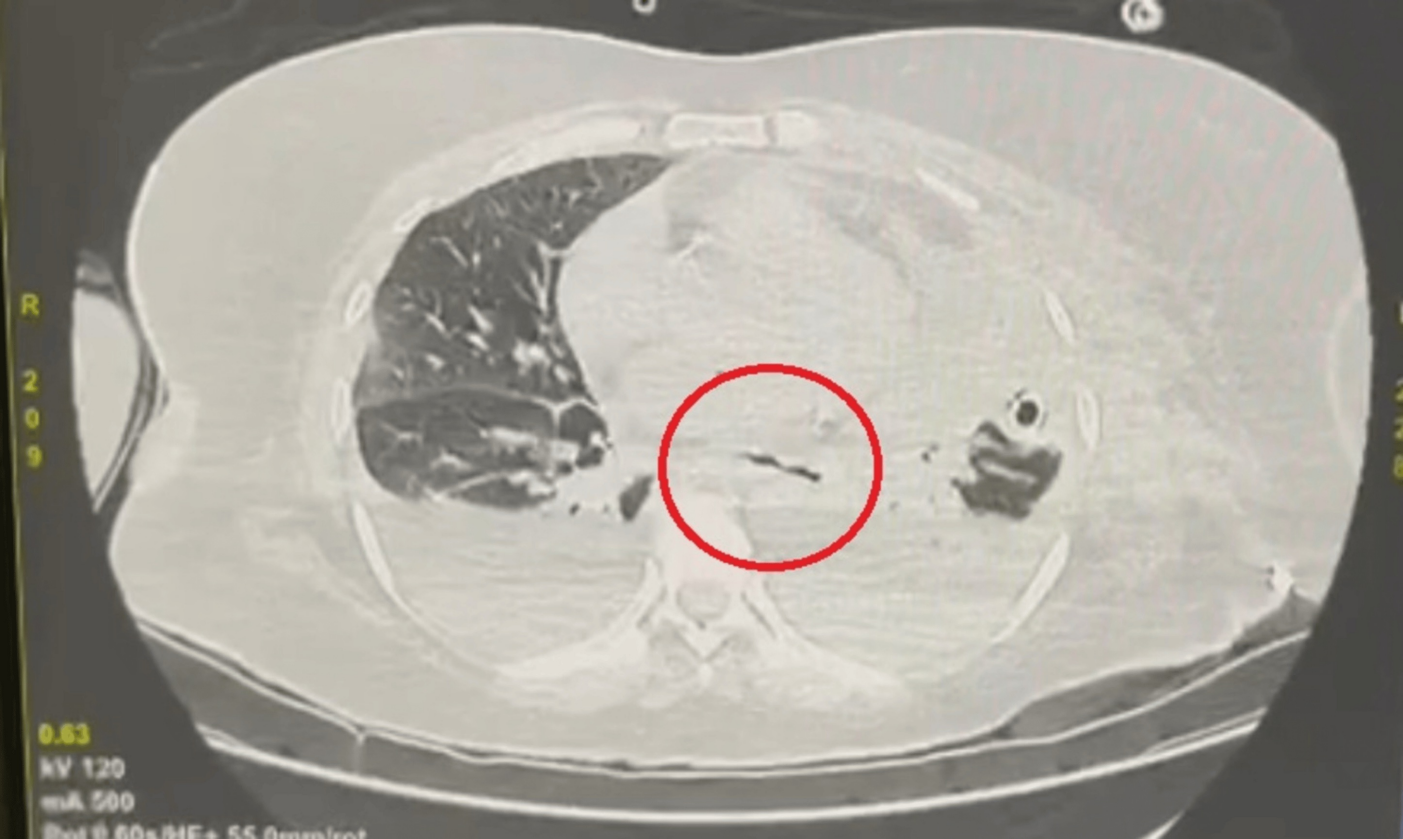
Contrast-enhanced computed tomography of the chest showing the esophageal rupture and leak into the left pleural space and mediastinum

CECT revealed characteristic features indicative of Boerhaave's syndrome, including pneumomediastinum and contrast extravasation from the distal thoracic esophagus. A perforation of approximately 3 cm in length was identified on the left anterolateral aspect of the thoracic esophagus, accompanied by left-sided pleural effusion (Figure 2).

**Figure 3:**
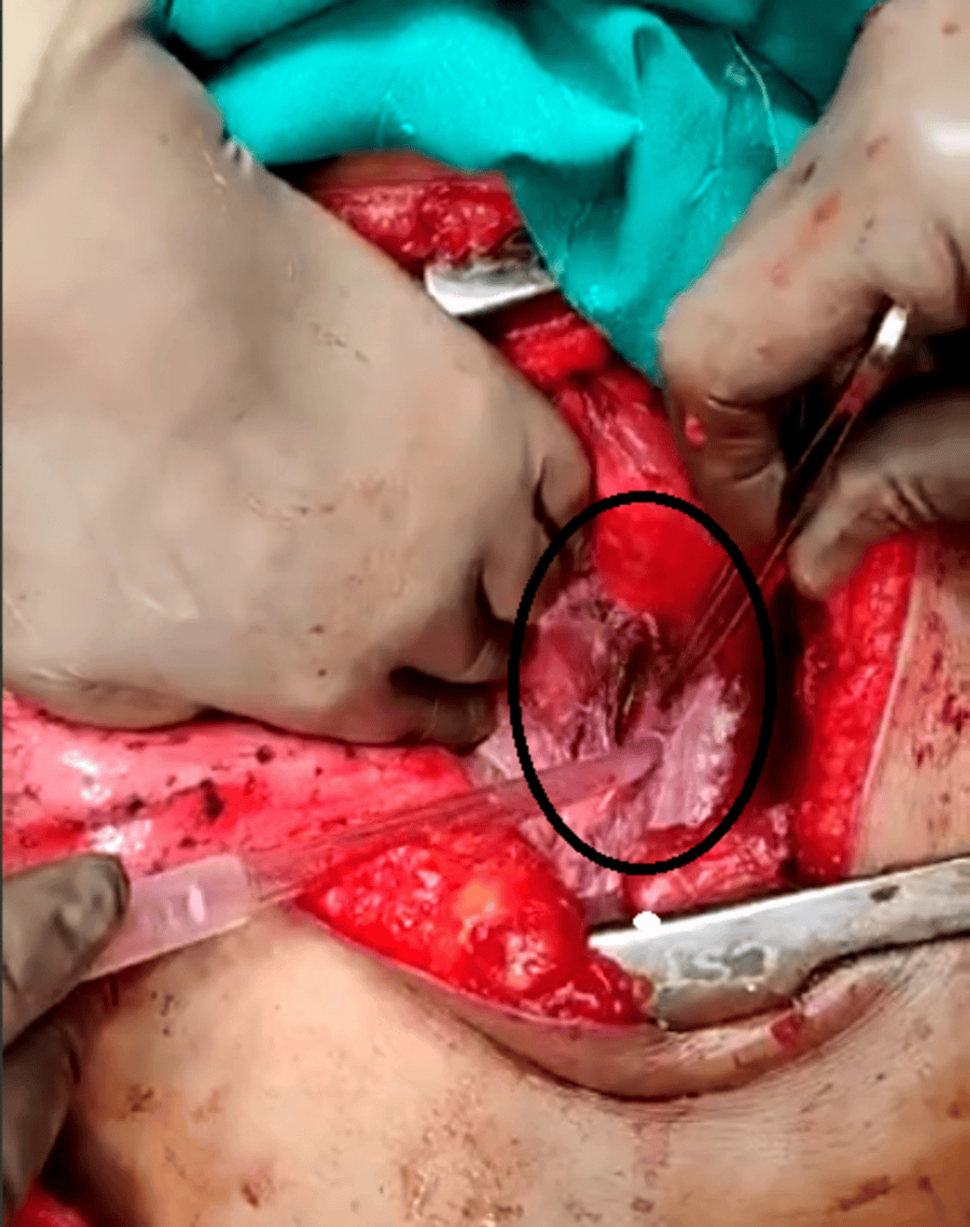
Intraoperative image of a full thickness tear in the thoracic esophagus

This imaging substantiated our diagnosis, prompting an immediate transfer of the patient to the surgical intensive care unit for stabilization and definitive management. An intercostal drainage tube was inserted on the left side to drain large amounts of fluid with debris. Following initial stabilization, the patient underwent emergency thoracotomy through a left anterolateral incision with laparotomy and exploration, revealing a longitudinal tear in the mid-esophagus approximately 3.5 cm in length, along with posterior mediastinal necrosis consistent with Boerhaave's syndrome (Figure 3).

**Figure 4:**
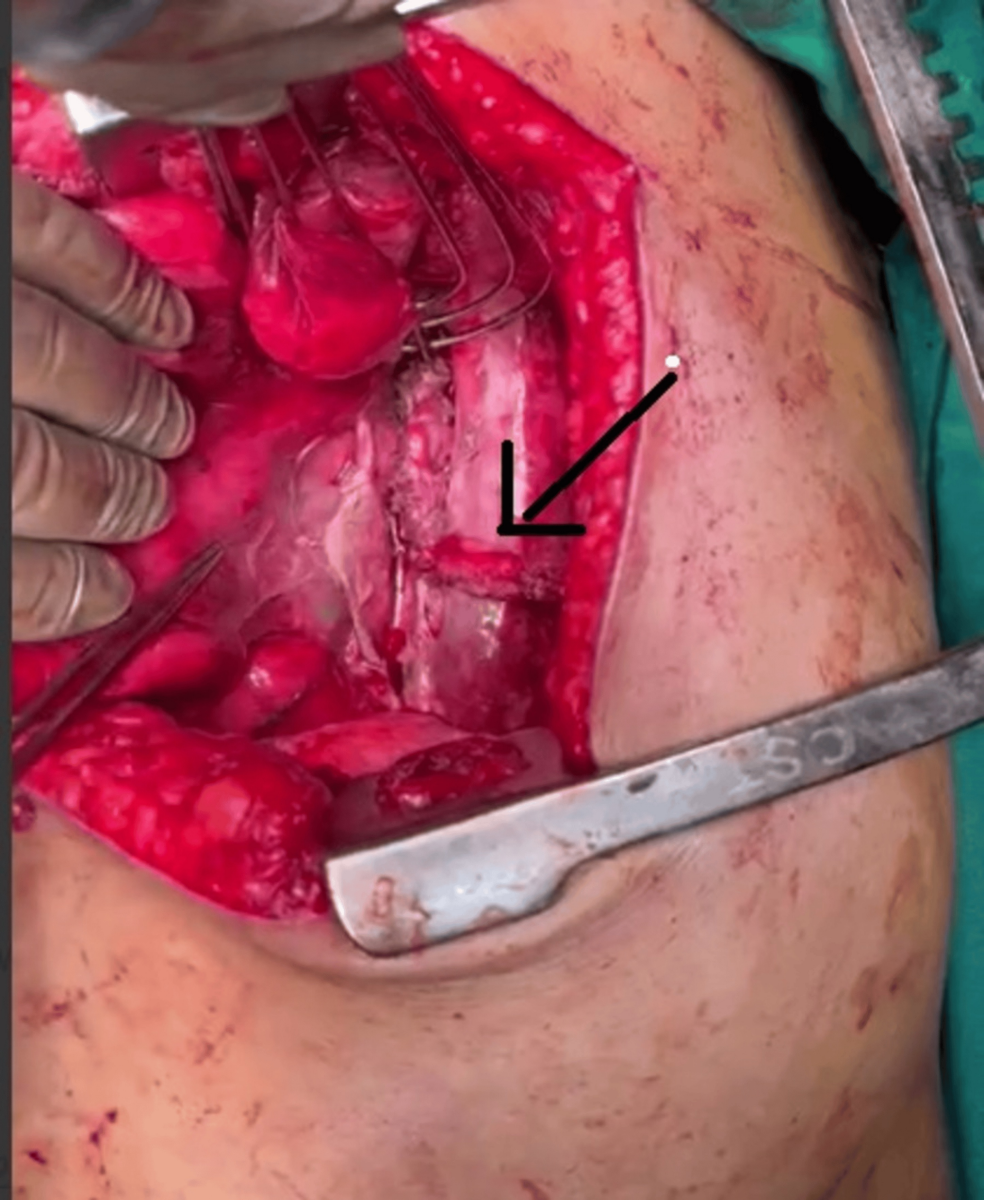
Intraoperative image showing the intercostal muscle flap to reinforce the primary repair (black arrow)

Debridement of the necrotic tissue and the margins of the esophageal tear was performed, followed by primary repair with polypropylene suture 2-0. The repair was reinforced with an intercostal muscle flap from the fifth intercostal space (Figure 4).

A nasogastric tube was passed beyond the site of rupture, and a defunctioning gastrostomy with feeding jejunostomy was performed as the patient was unwilling to have a defunctioning esophagotomy preoperatively. Thorough irrigation was administered through the intercostal drain, which was replaced intraoperatively.

**Figure 5:**
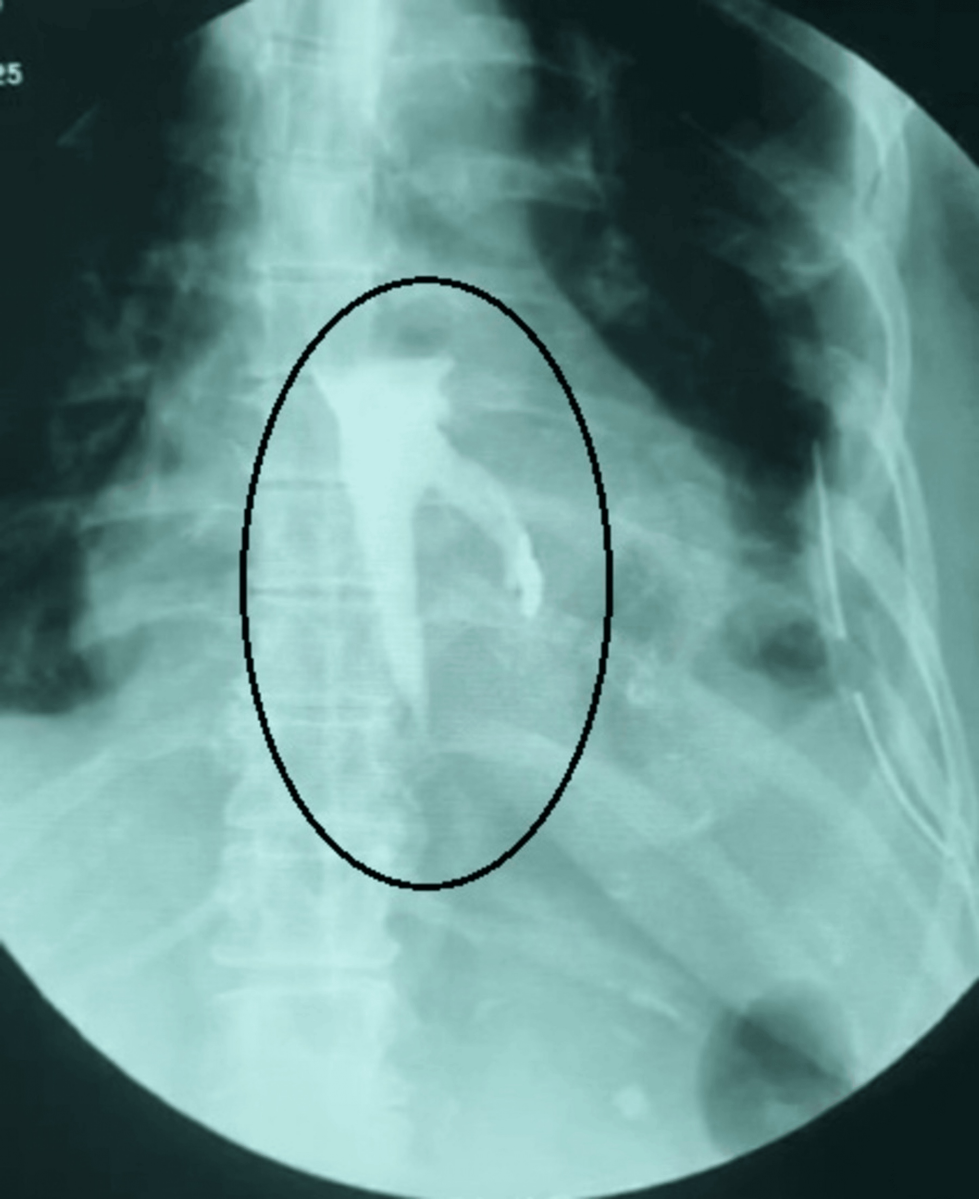
Esophagogram showing leak of contrast into the mediastinum on the left side

Postoperatively, the patient was closely monitored for signs of complications, including sepsis, respiratory compromise, and recurrent leakage. On postoperative day 7, increased intercostal drain volume coupled with tachycardia raised suspicion of a recurrent leak from the perforation site. An esophagogram revealed leakage of contrast fluid into the mediastinum with loculation (Figure 5).

A mini-thoracotomy was performed under local anesthesia to drain the collection, and the intercostal drain tube was changed after thorough washes. The patient was continued on feeding jejunostomy for six weeks, and her intercostal drain output and gastrostomy output were monitored simultaneously. Another esophagogram done after six weeks showed a minimal leak. In view of old age and comorbidities, along with a decrease in leak, the patient was continued on conservative management.

Regular monitoring ensued, with the patient maintained nil per oral for up to 12 weeks while receiving nutritional supplementation through the feeding jejunostomy. A repeat computed tomography after 12 weeks demonstrated resolution of the esophageal leak. The intercostal drainage tube and the gastrostomy tube were removed after 12 weeks once the patient was started on oral sips followed by a liquid diet. The patient was gradually transitioned to a soft diet, then subsequently to a full oral diet, and had the feeding jejunostomy tube removed after 14 weeks. A one-year follow-up has shown her to be disease- and symptom-free.

## Discussion

Boerhaave's syndrome is a rare yet potentially life-threatening condition that necessitates swift diagnosis and intervention to mitigate the risks of severe complications and mortality. It is a result of barotrauma where the intra-esophageal pressure may increase up to 200 mmHg [1]. While the classic Meckler’s triad of vomiting, lower thoracic pain, and subcutaneous emphysema is observed in only a minority of cases, its absence underscores the need for clinical suspicion and vigilant assessment due to the diagnostic complexities involved [2]. In this regard, utilizing imaging modalities such as CT with oral contrast or esophagography plays a pivotal role in facilitating a timely diagnosis [3]. In this patient, who presented with vomiting and chest pain, a cardiac event was ruled out pertaining to normal ECG findings, and subsequent development of a subcutaneous emphysema prompted a diagnosis of Boerhaave's syndrome.

Early surgical intervention stands as the cornerstone of treatment for Boerhaave's syndrome, with procedures like primary repair or esophageal diversion techniques serving as the primary approaches for management [4]. The typical approach for treating Boerhaave's syndrome involves repairing the esophageal tear as a primary procedure; however, studies show the use of intercostal muscle flap, latissimus dorsi flap, and in some cases diaphragmatic flap to reinforce the primary repair [5,6]. In the present case, an intraoperative decision was made to reinforce the primary repair with an intercostal muscle flap. Despite advancements in surgical techniques and critical care, the mortality rate associated with Boerhaave's syndrome remains significant, with higher mortality in cases who are referred late to a tertiary care center or those managed conservatively at the time of presentation [5]. This emphasizes the ongoing necessity for multidisciplinary management strategies and continued vigilance to optimize patient outcomes.

Various minimally invasive techniques, including video-assisted thoracoscopic repair, self-expanding metallic stents, and vacuum therapy, can be selectively tried based on patient condition and presence of experienced surgeons in the treatment center.

In essence, Boerhaave's syndrome demands a comprehensive and collaborative approach from healthcare professionals to promptly identify and address the condition's complexities, thereby minimizing the risks and maximizing the chances of a favorable outcome for affected individuals [7].

## Conclusions

Boerhaave's syndrome is a rare but potentially fatal condition characterized by spontaneous esophageal rupture, often presenting with nonspecific symptoms such as chest pain and vomiting. Early recognition and prompt surgical intervention are paramount in mitigating associated morbidity and mortality. This case highlights that proper nutrition, diversion of gastric secretions, and control of sepsis are key principles in the management of such cases. Multidisciplinary collaboration involving emergency medicine, surgery, and critical care is essential for the optimal management of this challenging condition.
